# Attention-driven UNet enhancement for accurate segmentation of bacterial spore outgrowth in microscopy images

**DOI:** 10.1038/s41598-025-05900-6

**Published:** 2025-06-20

**Authors:** Saqib Qamar, Dmitry Malyshev, Rasmus Öberg, Daniel P. G. Nilsson, Magnus Andersson

**Affiliations:** 1 Department of Physics, Umeå University, 90187 Umeå, Sweden; 2 Integrated Science Lab, Umeå University, 90187 Umeå, Sweden; 3 Umeå Centre for Microbial Research (UCMR), Umeå University, 90187 Umeå, Sweden; 4 Faculty of Computing and IT, Sohar University, 311 Sohar, Oman

**Keywords:** Spores, Deep learning, Contamination, Image processing, Computational science

## Abstract

Analyzing microscopy images of large growing cell samples using traditional methods is a complex and time-consuming process. In this work, we have developed an attention-driven UNet-enhanced model using deep learning techniques to efficiently quantify the position, area, and circularity of bacterial spores and vegetative cells from images containing more than 10,000 bacterial cells. Our attention-driven UNet algorithm has an accuracy of 96%, precision of 82%, sensitivity of 81%, and specificity of 98%. Therefore, it can segment cells at a level comparable to manual annotation. We demonstrate the efficacy of this model by applying it to a live-dead decontamination assay. The model is provided in three formats: Python code, a Binder that operates within a web browser without needing installation, and a Flask Web application for local use.

## Introduction

Light microscopy is the most widely used tool for visually characterizing bacterial cells and their behavior. One of its advantages over other imaging techniques is that it allows for imaging and monitoring of live cells, including cell growth, division, and death, with enough resolution to see sub-cellular features^1^. This is an advantage over bulk studies, which do not provide individual insights into the behavior of cells, and over electron microscopy, which works with fixed (dead) cells. Additionally, when studying bacteria it is important to have a large representative sample, which can sometimes number in the thousands of cells, especially when comparing the efficacy of decontamination and biocides^2–4^. However, tracking individual cells in a large sample over time, when using timelapse microscopy, is difficult^5^. Consequently, researchers face a trade-off between the sample’s scale and complexity and the temporal constraints of the experiment^6^. This is particularly problematic when studying large samples of growing bacterial cells.

To address these challenges, recent years have seen the development of various computational tools. These tools facilitate and expedite the segmentation of microscopy images, elevating analysis to a computational level^7^. They are particularly useful in analysing small features like flagella^8^, analysing 3D timelapse images^9^ or cell phenotyping^10^. Meanwhile, tools like SporeTracker are specifically designed to track germination^11^. However, SporeTracker uses thresholding to segment spores and cell growth, which can limit its accuracy in distinguishing overlapping cells or handling heterogeneous image conditions. In recent years, there has been an increase in the use of machine learning (ML) and deep learning (DL) tools for segmenting or classifying cells and bacteria in microscopy images^12–16^. Ilastik^12^ employs a machine learning approach to train a random forest classifier, which then segments and classifies the image. DeepCell Kiosk^13^ is a cloud-native platform designed to leverage deep learning for cellular image analysis. Cellpose^14^ is a deep learning-based segmentation tool trained on a large and diverse dataset of cell images. It uses a convolutional neural network (CNN) to perform segmentation without the need for retraining or parameter adjustment. Omnipose^15^ improves upon Cellpose by accurately segmenting samples with significant morphological variability, such as mixed bacterial cultures and antibiotic-treated cells, using unique CNN-based network outputs like the gradient of the distance field. Despite their strengths, these methods face limitations when applied to diverse cell types because they are not specialized models for specific data. A common deep neural network architecture well-suited for image segmentation is UNet^17^. UNet has been shown to have remarkable performance for image segmentation^18–20^. The name UNet comes from its U-shaped architecture, which has a contracting path to gather context and a symmetric expanding path for precise localization. It uses convolutional layers to extract image features and pooling layers to downsample feature maps. UNet connects the contracting and expanding paths’ layers of equal resolution via skip connections, enabling it to retrieve fine-grained spatial information that may have been lost during downsampling. A key advantage of UNet is its use of multiscale feature maps, allowing the network to capture objects of various sizes, which can be important for segmentation. However, UNet struggles with segmenting small structures in images^21^.

To improve UNet’s limitations in segmenting both small and large structures in an image, attention mechanisms can be integrated. These mechanisms are specifically designed to focus on the target object in an image^22^. Attentive weighting allows focusing on the key parts of the representation while filtering unnecessary elements. The weighted combination of channels then serves as an input to the first fusion stage, thereby improving the information flow in the decoder pathway. Recent studies have demonstrated that incorporating attention in UNet can significantly improve performance for a given task^23,24^. Attention mechanisms serve as a crucial tool in enhancing desired signals while minimizing unwanted noise, thereby directing the network’s focus towards the most relevant features for a given task^25^. Oktay et al^26^ incorporated attention gates into the UNet model for medical image segmentation, significantly improving performance on the task of pancreas segmentation. Similarly, Schlemper et al.^27^ used spatial attention mechanisms to enhance the segmentation of cardiac structures in MRI images. These studies highlight the potential of attention mechanisms to address the limitations of UNet in handling small and complex structures. This capability is particularly advantageous in the context of imaging microbiological systems, which are characterized by high signal variance and the presence of elements that can complicate segmentation^28^. Bacterial spores are, in particular, complicated to segment given their changes in brightfield optical density during spore germination (CaDPA release) and significant morphological alterations during the outgrowth phase^29,30^. Thus, both large and small features are present that change over time. This underscores the need for sophisticated segmentation methods to adapt to and effectively manage these complexities.

**Fig. 1:**
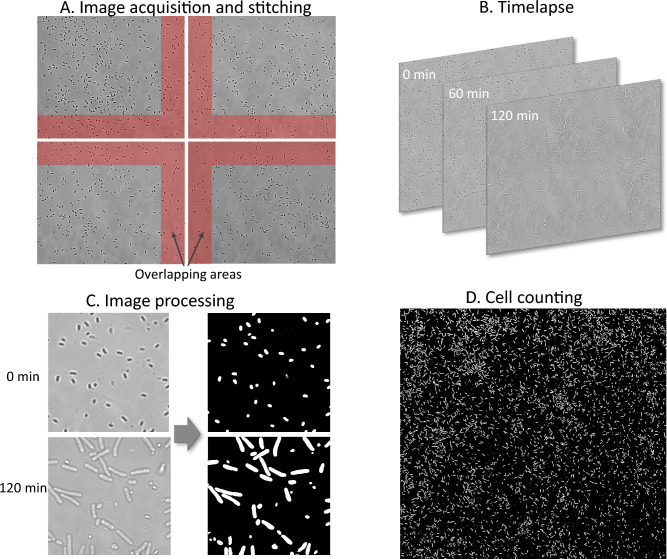
The cell segmentation and counting workflow. (**A**) The spores are imaged, with multiple overlapping fields of view to form a multi-image grid. The images are then stitched together into a composite image, which can contain >10,000 bacterial spores. (**B**) The process involves repeating the imaging to allow viable spores to germinate in nutrient media. A timelapse is obtained as a result of this. (**C**) The proposed UNet algorithm processes composite images to provide a binary mask of spores and germinated cells. (**D**) Free software tools such as ImageJ can be used to count cells and spores from binary masks. Filtering spores from vegetative cells can be done by knowing their area and circularity.

In this work, we develop and test the performance of an attention mechanism incorporated into a UNet architecture to enable the analysis of microscope images containing over 10,000 bacterial spores that germinate (“spore-UNet”). We also present a workflow to automatically image and combine multiple fields of view images of spores deposited onto a surface, see Fig. 1. Composite images are analysed using spore-UNet to produce an accurate mask, identifying individual spores and cells. This approach offers opportunities for direct spore and bacteria quantification and monitoring growth. We demonstrate this by conducting a live-dead assay where we monitor the germination and outgrowth after decontamination.

## Materials and methods

### Study setup and evaluation

In this section, we will explain in detail the method we propose for segmentation. During the training phase, we include normalization and contrast enhancement in the preprocessing step. To increase the number of training samples, we slice the original image into smaller patches and augment them to feed into the network’s input.

#### Pre-processing

Microscopy images often have quality issues that can make analysis difficult. Some common problems include uneven brightness across the image, lack of contrast between the features of interest, and visual artifacts such as debris or staining imperfections. These issues can reduce the accuracy of models that attempt to analyze these images. To minimize these problems, we first pre-processed the images by normalizing the brightness. The initial pre-processing step was to normalize the brightness of the spore images. This helps to account for differences in the overall brightness from one image to the next.

We normalized each image according to Eq. 1 below:

1 $$\begin{aligned} I_{norm} = \frac{I - \mu }{\sigma } \end{aligned}$$

where *I* is the input image, $\upmu$ is the mean intensity, and $\sigma$ is the standard deviation of intensity. Once normalization is done, contrast enhancement is applied. Contrast enhancement helps improve the visual quality of images by stretching the range of intensity values they contain. For contrast enhancement, histogram equalization is used. The histogram equalization transform function is according to Eq. 2 below:

2 $$\begin{aligned} T(r) = \sum _{k=0}^r p(r_k) \end{aligned}$$

where *T*(*r*) is the transform function that maps the original grayscale pixel value *r* to an enhanced value. $p(r_k)$ represents the probability of occurrence of a pixel with intensity value $r_k$. The function of $p(r_k)$ can be written as

$$\begin{aligned} p(r_k) = \frac{n_k}{N} \end{aligned}$$

where $n_k$ is the number of pixels with intensity value $r_k$ and *N* is the total number of pixels in the image. The combined effect of normalization and histogram equalization contributed to a higher input quality for training. We observed that normalization improved the consistency of histogram equalization results, especially when processing batches of images with varying lighting conditions.

#### UNet based architecture

**Fig. 2:**
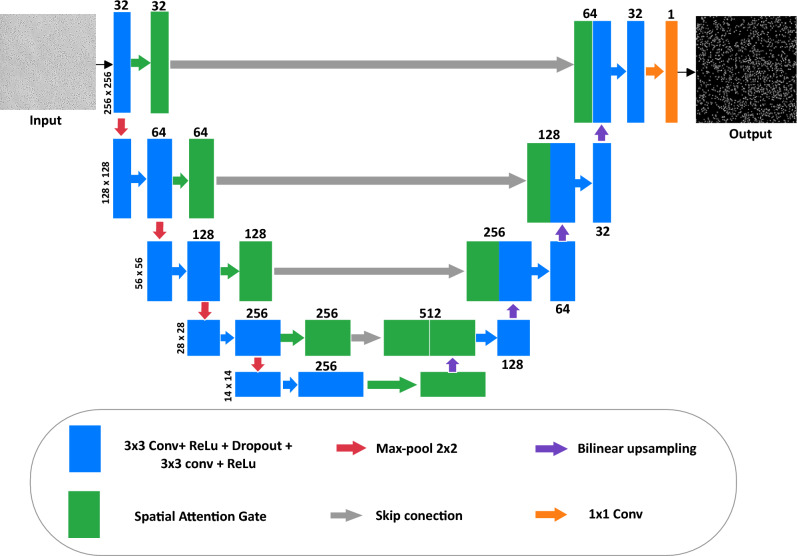
An example input passed through our spore-UNet model (color version optimal). The rectangular bars show multi-channel feature maps extracted at each layer. The numbers above the bars indicate the number of channels, whereas the vertical digits on the left display the x-y dimensions of the feature maps.

The spore-UNet model modifies and enhances the standard UNet architecture. As shown in Fig. 2, UNet has a U-shaped encoder-decoder structure. On the encoder side (left half), the process involves repeated max pooling to reduce image size by half, coupled with double convolution operations that increase feature maps twofold. The decoder side (right half) mirrors the encoders, reversing the downsampling and feature-mapping steps. Similar to the original UNet, spore-UNet uses four encoder-decoder modules connected in this manner.

Each decoder module has three main stages: bilinear up-sampling to double the spatial dimensions of the feature maps (violet arrows). Next, we concatenate these upsampled features with the outputs from the corresponding encoder module (gray arrows). This allows multiscale information to flow through the network. The third is double convolution, which halves the number of feature maps and condenses information. Skip connections enable the combination of hierarchical features from the encoder side for more contextual output. Finally, the last layer of spore-UNet applies a 1 $\times$ 1 convolution (brown arrow) to produce a single feature map to predict output segmentation. The decoders upsample the features, fuse them with encoder outputs, transform the combined representation through convolution operations, and ultimately generate pixel-wise classifications. This decoding phase restores the spatial resolution while benefiting from the encoder context.

The spore-UNet model modifies the original UNet architecture by adding attention modules to the encoder pathway. Specifically, we employed channel and spatial attention blocks after the first convolution and each subsequent encoder (green arrows in Fig. 2). This amplifies informative features and suppresses less useful features at each image scale. Importantly, the encoder attention modules, such as convolution, do not directly process the input image. Rather, they are only fed into the decoders through skip connections to retain all original image details up to the bottleneck. This selective highlighting of encoder features guides the decoder to refine and transform these representations for accurate final segmentation.

#### Spatial attention gate

The spatial attention gate is a key enhancement to the standard U-Net architecture. Introduced in the Attention U-Net design, it selectively emphasizes salient encoder features before they are passed through skip connections to the decoder. In this configuration, each attention gate receives feature maps from the encoder and a corresponding gating signal from the decoder, allowing the model to learn spatially selective masks that suppress irrelevant background activations and highlight semantically meaningful regions. This is particularly valuable in grayscale images, where critical structures often occupy small areas with low contrast.

**Fig. 3:**
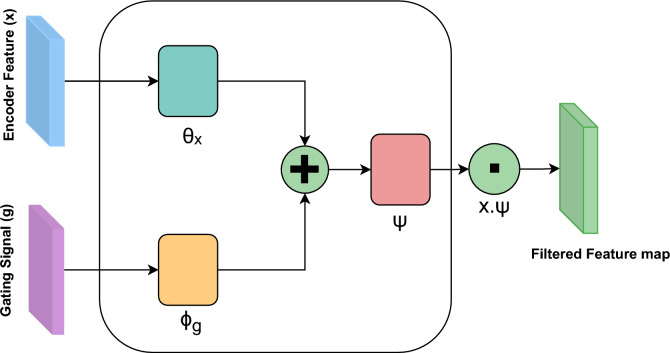
Schematic of the Spatial Attention Gate. The encoder feature *x* is compressed to $\theta _x$, and the decoder signal *g* is projected and upsampled to $\phi _g$. Their sum is used to produce the attention map $\psi$, which is broadcast and multiplied by *x* to generate the filtered feature map.

To compute the spatial attention mask, an encoder feature map $x \in \mathbb {R}^{H \times W \times C_x}$ is first downsampled using a $2 \times 2$ convolution with stride 2, producing a compressed representation $\theta _x \in \mathbb {R}^{\frac{H}{2} \times \frac{W}{2} \times C_{\text {int}}}$. Simultaneously, a gating signal from the decoder $g \in \mathbb {R}^{\frac{H}{2} \times \frac{W}{2} \times C_g}$ is processed using a $1 \times 1$ convolution and upsampled to match the spatial dimensions of $\theta _x$, yielding $\phi _g$. A schematic of the process is shown in Fig. 3.

The attention coefficients are computed by summing $\theta _x$ and $\phi _g$ element-wise, followed by a ReLU activation to produce an intermediate map *f*. This is then processed using a convolution $1 \times 1$ and a sigmoid function to generate the spatial attention map $\psi \in \mathbb {R}^{\frac{H}{2} \times \frac{W}{2} \times 1}$. This map is upsampled to the resolution of the original input *x*, repeated along the channel axis to match $C_x$, and multiplied element-wise with the encoder feature map to produce the filtered feature map $F_s$ as shown in Eq. 3.

3 $$\begin{aligned} F_s = x \cdot \text {Repeat}(\text {Upsample}(\psi ), C_x) \end{aligned}$$

A final convolution $1 \times 1$ followed by batch normalization is applied to restore the feature map to its original shape. This spatial attention mechanism ensures that only the most relevant encoder features are forwarded through skip connections, improving the model’s ability to localize fine structures and suppress irrelevant context in segmentation tasks.

### Training, validation, and testing dataset

For imaging spores, we used an inverted microscope (Olympus IX71, Olympus) equipped with a temperature-controlled water immersion objective (UPlanSApo60XWIR 60X N.A. = 1.2; Olympus) and a servo-controlled sample stage^31,32^. Together with the microscope, we used a 1920 × 1440 pixel CMOS camera (C11440-10C, Hamamatsu) to record brightfield images (1920 × 1440 pixels each).

For training, we prepared a dataset consisting of 8285 images to train spore-UNet. The dataset comprises 7000 training images, 1000 validation images, and 285 testing images. We generated smaller 256 × 256 patches from the camera output images (1920 × 1440 pixels) to use as input to the model and save memory. During the patch creation process, we only included patches that had more than 5% of patch area occupied by data points (bacterial cells). The dataset has both the original images and their ground truth labels. We also augmented the training data by rotating, scaling, and flipping images to create more variety. Data augmentation helps improve the machine learning model performance by generating new and varied training examples^33^. With larger, comprehensive training datasets, the model will be more accurate and identify accurate boundaries of segmented objects. Thus, this helps the model learn robust features that generalize better. During training, the training and validation datasets are used to tune the model hyperparameters. To evaluate the model, we used test data, which was unseen during training. We built the model architecture in Python 3 using Keras 2.7 and ran experiments on an NVIDIA GeForce RTX 3070 graphics card.

### Annotation of images

Image annotation is the process of labeling where objects of interest are located in images. This helps deep learning models recognize and classify objects. We annotated our dataset using the Roboflow application^34^, a web-based annotation tool that requires no complex setup (Fig. S1). Although Roboflow is used for the initial region of interest, the resulting annotations are not used directly. Instead, all masks are manually refined using polygon points to ensure high-quality ground-truth labels. This correction process is essential, as the SAM-based segmentation in Roboflow frequently fails to resolve the morphological features of densely packed spores. We defined labels to provide information about what is shown in each image. We used polygons to outline the boundaries of the spores and the grown bacteria (Fig. S2). The annotation results were stored in a JSON file with x and y coordinates and labels. Finally, we converted these polygon points into binary mask images for each object. Thus, annotation prepares image data to train deep learning models to detect and classify objects of interest. We annotated a total of 724 images, and these are used to create more patches for training data.

### Statistical metrics used for evaluation

We assessed the performance of the model using standard machine learning metrics. We used five metrics, such as accuracy, precision, sensitivity, specificity, and F1-score, to evaluate the model’s performance. These metrics are computed by comparing the predicted labels of the model for each pixel against the true labels in the reference data. They are calculated based on the number of true positive (TP), true negative (TN), false positive (FP), and false negative (FN) predictions made by the model^35^. We will display the mathematical definition of each item mentioned below.

Accuracy measures the proportion of correct predictions made by a model out of total predictions.

$$\begin{aligned} Accuracy = \frac{(TP + TN)}{(TP + TN + FP + FN)} \end{aligned}$$

Precision measures the proportion of positive predictions made by the model that are correct.

$$\begin{aligned} Precision = \frac{TP}{(TP + FP)} \end{aligned}$$

Sensitivity measures the proportion of actual positive samples correctly identified by the model.

$$\begin{aligned} Sensitivity = \frac{TP}{(TP + FN)} \end{aligned}$$

Specificity measures the proportion of actual negative samples that the model correctly identifies.

$$\begin{aligned} Specificity = \frac{TN}{(TN + FP)} \end{aligned}$$

The F1-score is the harmonic mean of precision and sensitivity. It considers both FP and FN predictions. A model with a high F1 score has high precision and sensitivity.

$$\begin{aligned} F1-score= \frac{2*TP}{(2*TP + FP + FN)} \end{aligned}$$

Intersection over Union (IoU) measures the overlap between the predicted and ground truth regions. It is defined as the ratio of the intersection to the union of the predicted and true positives.

$$\begin{aligned} IOU = \frac{TP}{(TP + FP + FN} \end{aligned}$$

These results were then collected into a confusion matrix. The diagonal cell of the confusion matrix represents the correctly classified instances for a class. By analyzing the confusion matrix, we could identify the strengths and weaknesses of the spore-UNet model.

### Cell counting experiment

#### Spore preparation

*Bacillus thuringiensis* *(B. thuringiensis)* ATCC 35646 cells were grown on BBLK sporulation agar (BD) plates, incubated at 30 °C overnight^36^. Cells were collected by scraping them off the agar and transferred to a 1.5 mL Eppendorf tube, centrifuged once with the supernatant discarded to remove leftover growth media and the pellet resuspended in deionised water. To allow sporulation, the cells were stored overnight at 4 °C. The resulting spore suspension was then purified by centrifuging in deionised water at 5000 $\times$ g for 5 min five times, discarding the supernatant and resuspending the pellet each time. After being purified, the suspension was resuspended in deionised water and stored at 4 °C.

The sample was prepared by depositing 5 $\upmu$L of a 10^7^ spores/mL suspension on a glass cover slip (no.1, Paul Marienfeld GmbH & Co., Lauda-Königshofen, Germany) and allowing it to dry to fix the spores to the surface. To observe the process of spore germination and growth, a 5 $\upmu$L drop of tryptic soy broth (TSB, Bacto^TM^, BD) was added on top of the dried spores and the sample was sealed by placing a vacuum grease ring around the drop and covering it with a 20 × 20 mm cover slip. The sample was then kept at 30 °C, while acquiring images of a 500 × 500 $\upmu$m sample area.

#### Image stitching

The image acquisition for stitching was done using an in-house developed LabView program for controlling the sample stage and the camera (see SI section “Image Acquisition in LabView”). The program uses a snake-by-rows algorithm to capture 49 overlapping fields of view in a 7 $\times$ 7 grid. The sequence takes less than 1 minute to complete and the process was repeated every 20 min, resulting in a timelapse series of composite images. To create the composite images, each image sequence was combined in Fiji (ImageJ, 1.53t)^37^ using the image stitching plugin^38^. The 49 fields of view were merged into a combined image (stitched) that covered a 500 × 500 $\upmu$m area with a resolution of approximately 16.5 pixels/$\upmu$m. The supplementary material includes the LabView code and a step-by-step guide on how to perform these tasks.

#### Counting cells

We determined the number of cells using the free software Fiji (ImageJ) and the masks were processed using the “Analyze Particles” tool.

To determine typical spore and cell size and shape and thus filter noise and false positives, we compared spores on the UNet-generated masks with the corresponding original unprocessed images. We manually checked around 100 particles, measuring their size and circularity. Following this pre-measurement we have chosen the following filter parameters for the “Analyze Particles” tool settings: 0.2–0.95 for circularity, pixel size 50–750 for the 0 min time point (when the sample is only spores) and pixel size 50–5000 for the 120 min time point (when large cells are present). Particles outside these parameters were discarded. We manually assessed these filter settings.

## Results and discussions

### Model evaluation and validation

With the aim of developing an advanced machine learning model specifically tailored for the classification and analysis of spore images, we trained the spore-UNet model using 7000 spore images and validated the model using 10,00 images. The images were 256 $\times$ 256 pixels and contained gray channel information. To enhance the learning process, we employed Adam optimization, a training technique known for its efficient computation and effective handling of sparse gradients^39^. This allowed us to set a learning rate of 0.002, which determines how quickly the model learns from each batch of images. We also set two momentum parameters, $\beta _1$ and $\beta _2$, which help stabilize and speed the learning. The model was then trained for 50 epochs.

**Fig. 4:**
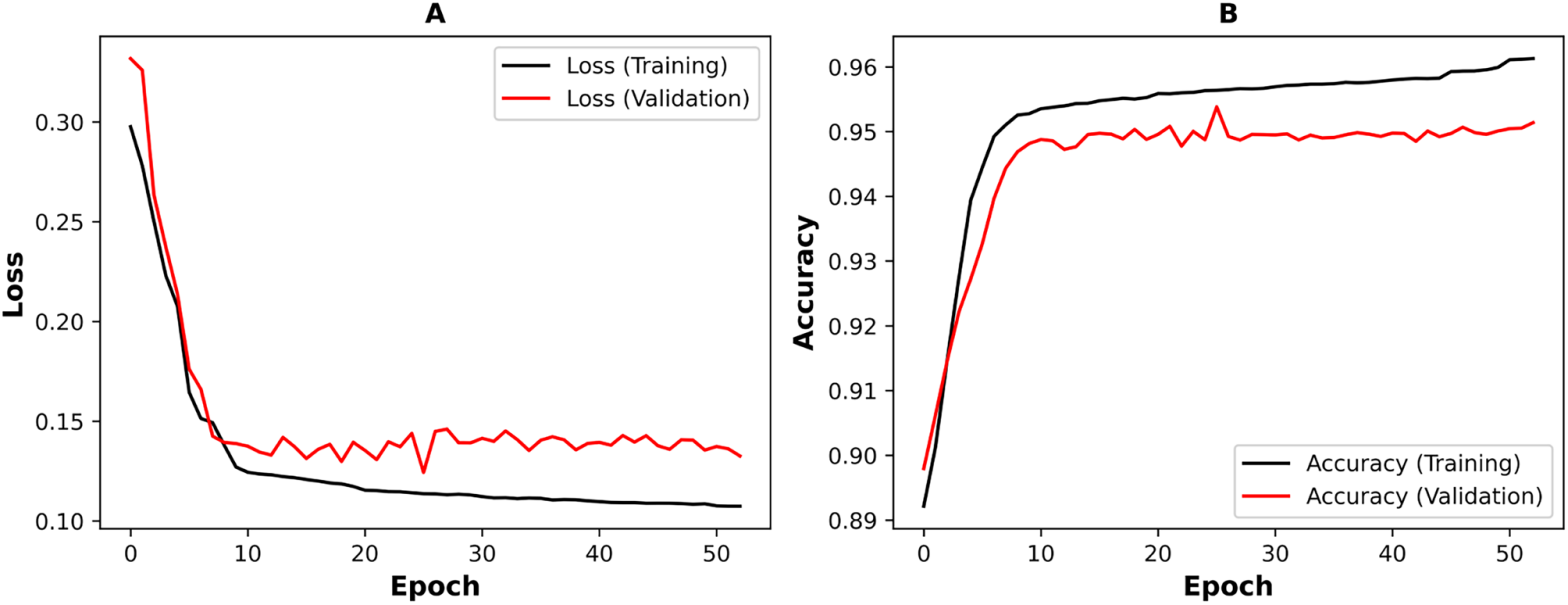
Training and validation metrics over Epochs. (**A**) illustrates the decrease in training loss across 50 epochs, demonstrating the model’s improving performance in learning from the dataset. (**B**) shows the increase in validation accuracy over the same 50 epochs, indicating the model’s enhanced ability to generalize and accurately classify new, unseen data. During the first 10 epochs, loss decreases and accuracy sharply rises, followed by a plateau. After 10 epochs, the training set accuracy continues to rise, but the validation accuracy remains largely unchanged.

**Fig. 5:**
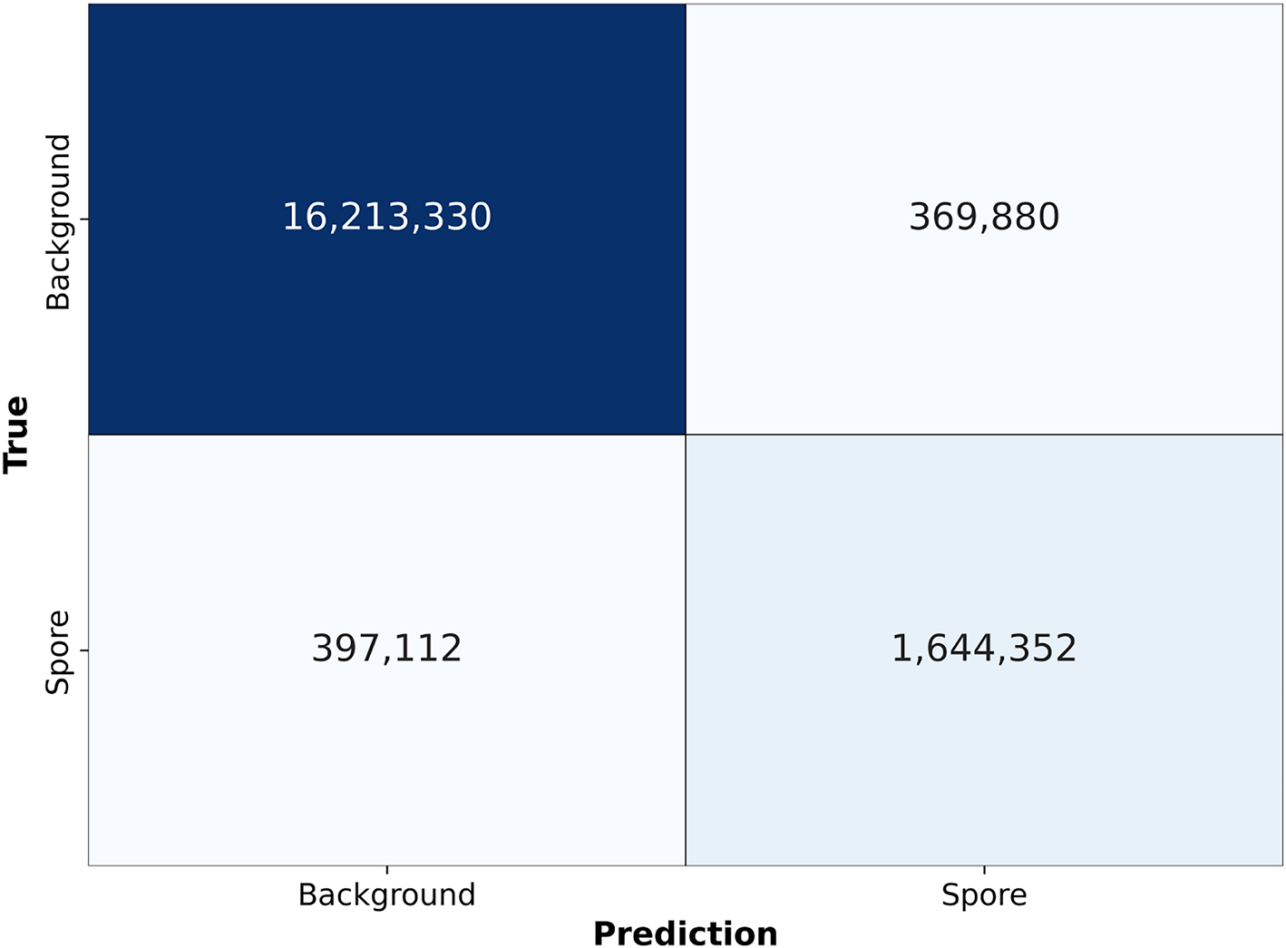
Confusion matrix demonstrates the pixel-level classification performance on the test set. The rows indicate the true class from the ground truth, whereas the columns show the predicted classes output by spore-UNet. The diagonal cells display correctly classified instances as either spores or backgrounds. The off-diagonal elements indicate misclassified examples.

After about 10 epochs, the model accuracy reached a plateau of around 95% for the validation set, as shown in Fig. 4. This means that the model could accurately classify the content of images that were not used during training. The loss curve also shows a similar downward trend, indicating that the model continued to improve. By the time it reached 50 epochs, the model had minimal additional learning, suggesting that it was well-trained. We used the confusion matrix for our data, shown on Fig. 5, to calculate the Precision, Sensitivity, Specificity, and F1-Score for each class in the data set. Based on these metrics, we concluded that the spore-UNet model performed well across all measures.

We use an Attention-based UNet architecture for spore segmentation. To assess its performance, we compared it with traditional UNet and residual-based UNet architectures for spore segmentation. We evaluated different UNets based on the Precision, Sensitivity, Specificity, F1-score, Accuracy, and Intersection over Union (IOU). Based on the test data, it can be concluded that the spore-UNet model has achieved and accuracy of 96%, precision of 82%, sensitivity of 81%, specificity of 98%, F1-score of 80%, and IOU of 71%. These results suggest that the modifications made to the UNet architecture have enhanced its ability to classify accurately and segment data with high consistency. The second-best performer among the other state-of-the-architectures was the Residual-based UNet model, which achieved 90% accuracy, 81% precision, 67% sensitivity, 94% specificity, 73% F1-score, and F1-score of 61% on the test data. In contrast, the UNet architecture used as a baseline achieved an accuracy of 88% on the test data.

We have validated the algorithm by comparing it to Cellpose, we were not able to segment test images accurately, achieving a precision of 78%, recall of 64%, accuracy of 70%, F1-score of 68%, specificity of 78%, and IOU of 0.54%. We believe this is due to the characteristics of our data, in which cells vary in size and shape, which pose challenges for optimization by Cellpose. We also run Omnipose with our dataset achieving a precision of 79%, recall of 67%, accuracy of 82%, F1 score of 73%, specificity of 78% and IOU of 0.57%. These improvements highlight Omnipose’s ability to better handle morphological variability. However, our spore-UNet still outperformed both models, demonstrating stronger generalization and robustness on spore-specific segmentation tasks.

Thus, we conclude that our experimental results suggest that the spore-UNet model has stronger robustness and higher generalization ability than other tested models for spore segmentation. For training, we used Roboflow just to mark the initial regions of interest in the images, but the final annotations were manually corrected using polygon points to make sure they were accurate and unbiased. So, our spore-UNet model did not learn from any pre-trained model. Instead, it was trained using expert-labeled data. Also, we found that general tools like Cellpose and Omnipose did not work well for our type of data. That’s why we developed a custom spore-UNet model. One of the main advantages of spore-UNet is that it can accurately segment a large number of bacterial cells, even when they look different or are very close together. This helps reduce both manual work and the need for retraining. The results of all models are listed in Table 1.

**Table 1:** Quantitative comparison of spore segmentation performance for different model architectures.

Method	Precision	Sensitivity	Specificity	F1-score	Accuracy	IOU
Cellpose	0.78	0.64	0.78	0.68	0.70	0.54
Omnipose	0.79	0.67	0.88	0.73	0.82	0.57
UNet	0.78	0.68	0.91	0.73	0.88	0.59
Res-UNet	0.81	0.67	0.94	0.73	0.90	0.61
spore-UNet	**0.82**	**0.81**	**0.98**	**0.80**	**0.96**	**0.71**

This table displays evaluation metrics measuring precision, sensitivity, specificity, F1-score, accuracy, and IOU. Spore-UNet demonstrates improved performance across all metrics compared to Cellpose, Omnipose, baseline UNet and Res-UNet. Largest values for each column are in bold.

### Segmenting spores in microscopy images

**Fig. 6:**
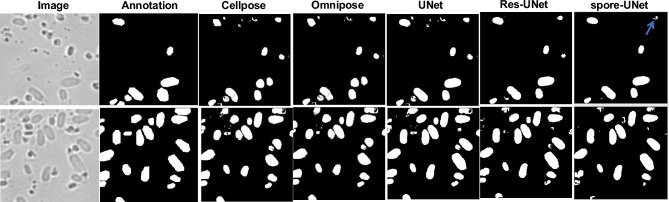
Comparison of model predictions for spore segmentation. The first column presents the original microscope images of spores and outgrown vegetative cells. The second column features the manual annotations that outline the spore and cell boundaries. The subsequent columns illustrate the performance of various algorithms: Cellpose, Omnipose, UNet, and Res-UNet, highlighting deviations from the manual annotations. The final column exhibits the segmentation predictions made by Spore-UNet, with the blue arrow indicating a false-positive region in the predictions.

To showcase the performance of the spore-UNet model in segmenting spores in microscopic images, we present examples in Figs. 6 and S2. Note that the spore samples were not cleaned using, for example, Histodenz or Nycodenz to remove debris, since we wanted to assess the model on complicated images. The original images are displayed on the left, and the manually annotated ground truth is provided in the middle for comparison. On the right-hand side, you can see the predicted segmentations generated by spore-UNet. Qualitatively, we see that our model can accurately delineate spore boundaries and internal textures. We evaluated the spatial alignment between predicted masks and the ground truth masks using the Intersection over Union metric, as presented in Table 1.

As evident from visual inspection, our model captured fine-grained edge details on the perimeter of each spore. Notably, the model properly segmented clustered spores. Compared to manual annotation, which segments spores and cells as relatively simple polygons (due to time limitations in manual annotation of $\sim$ 30,000 cells), the predictions (except the false positives) were in fact a better representation of the spores and cells in the raw data. This means that the limitations of manual annotation are a contributing factor to the lower performance metrics. The “real” prediction metrics would likely be higher if compared to a more finely annotated ground truth.

These capabilities highlight the proficiency of the model in identifying unique spore instances within each image. Validating the performance across our entire test set, we report an average segmentation accuracy of 95% compared to manual spore delineations. This high accuracy demonstrates the reliable generalization of our model to various spore morphologies and image backgrounds. While predictions appear largely correct, we observe false-positive regions, indicated by blue arrows. Incorporating more diverse training data with additional spore types would likely improve performance in these corner cases. Nonetheless, the current results confirm that our approach can segment spores at a level of accuracy on par with that of expert human labelers. Therefore, our model shows potential as an automated tool for analysing large images that contain a high quantity of spores.

### Quantifying germination and outgrowth of 10^4^ spores in large images

To demonstrate the use of the model for high-throughput analysis, we tracked the germination and outgrowth of *B. thuringiensis* spores on a large field of view. Spores are inactive seed-like forms of some bacteria that form when the bacteria are in unfavourable conditions. These spores are highly resilient to environmental challenges and disinfection procedures^40^ and thus some spore-forming species like *C. difficile* and *B. cereus* present a problem in healthcare^41^ and food processing^42^. Traditional methods of testing disinfection on surfaces are bulk testing, which results in losses of cells during the transfer from the surface to nutrient plates^43^. Direct imaging of the cells would avoid these losses and allow for a more representative assessment of the effect on the sample. However, typical disinfection requirements for spores on hard surfaces are 3–4 log^44^ and thus require at least 10^4^ cells to be counted, well outside what can be counted manually within a reasonable timeframe. However, counting 10^4^ cells falls within the capability of our workflow. As such, it can be used as a much more direct, accurate, and “real-world” way of assessing surface disinfection.

**Fig. 7:**
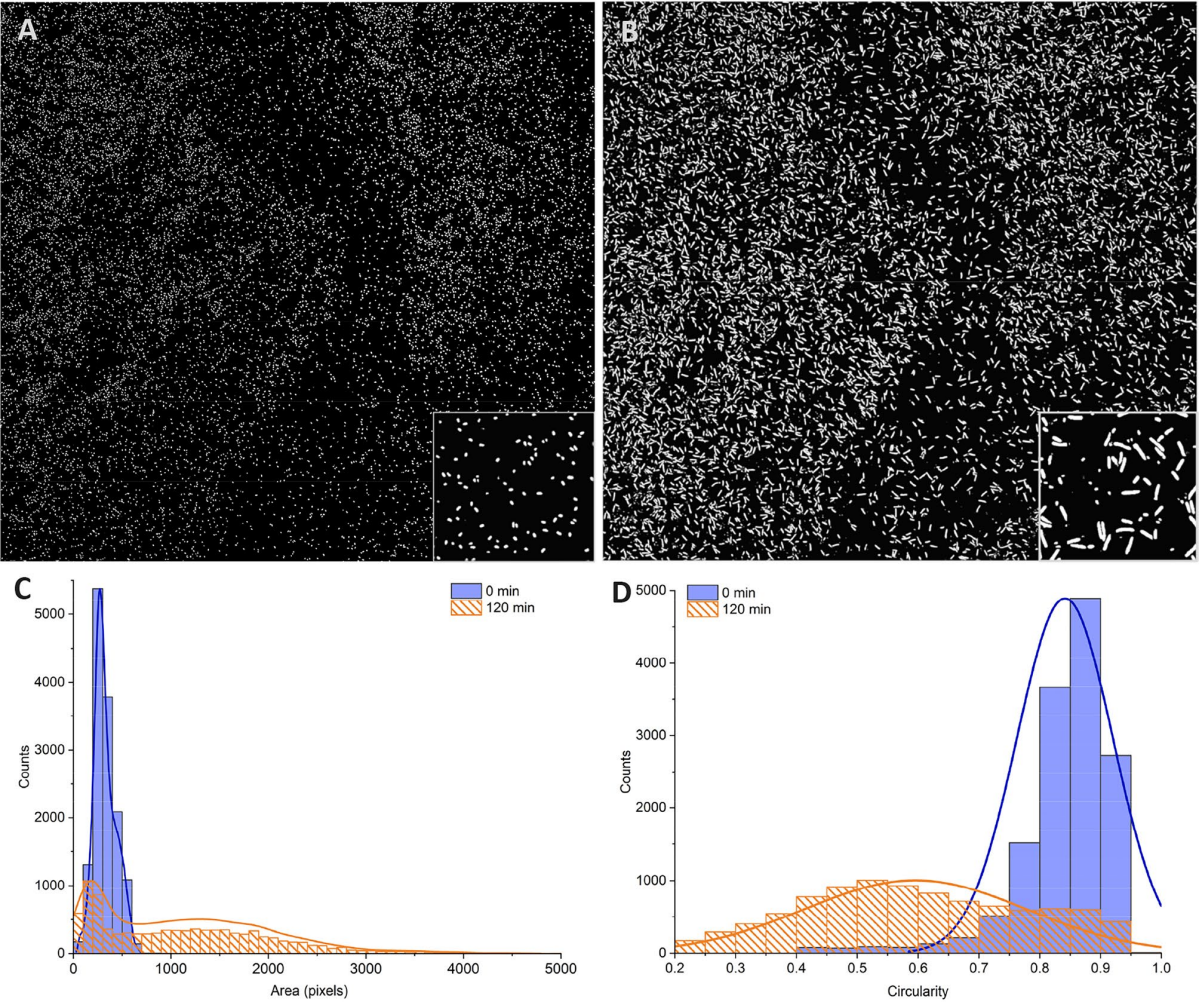
Tracking the germination and growth of *B. thuringiensis*. The algorithm segments spores both at the start of the measurement (**A**) and after 2 h (**B**), at which point, based on incubation duration and visual confirmation, spores have had the time to germinate and grow, but not yet divide. A higher resolution inset illustrates the segmented individual spores and cells. Particle analysis can be used to quantify differences in the sample over time, such as cell size difference (**C**) and circularity (**D**) to determine germination rates.

To simulate such a scenario, we placed spores on a glass slide, covered them with nutrient broth (TSB), and monitored their germination and growth for two hours. We then use the spore-UNet model described above to obtain binary masks for each time point, see Fig. 7A and B. From the masks, we extracted quantitative metrics (size, circularity) of the spores (Fig. 7A) and the germinated cells (Fig. 7B).

Counting spores and cells can be complex for automated tools, as the relative contrast of the spore can also be greatly affected by focus drift, as shown in Fig. S3. Additionally, as they grow out of the spore form, they increase in size, change their shape, as well as their optical density. However, we found that the spore-UNet model could resolve the cells despite these differences. The data indicate that spores exhibit a clear phenotype that depends on incubation time, as they germinate and grow into cells. More specifically, after filtering out artifacts with low pixel size, spores before nutrient incubation have a median area of 1.11 $\upmu$m^2^ with a 5–95% range of 0.6–1.97 $\upmu$m^2^ (n = 13972). These data exhibit a distribution that closely resembles a normal distribution, depicted in blue in Fig. 7C. In comparison, following 2 h of incubation, the median size of the particles is 3.73 $\upmu$m^2^ with a 5–95% range of 0.33–10.06 $\upmu$m^2^ (n = 9455), with a non-normal distribution (orange). The shape of the particles also changes, as shown by the circularity measurements shown in Fig. 7D. The spores before incubation show a narrow range of circularity (blue) with a median value of 0.86 and a 5–95% range of 0.71–0.93, compared to the incubated spores (orange), which have a median of 0.58 and a much wider 5–95% range of 0.30–0.90. One limiting factor for the algorithm can be dense overlapping cells, particularly when dividing. Cells can float away from the glass surface and overlap with each other, making counting difficult. A way to prevent this would be to use agar-based methods, such as sandwich agar pads. The cells are then limited to grow in a plane, preventing overlap. In experiments where this is not possible, the area of heavily overlapping cells can be excluded from analysis.

We have also manually examined particles that are outside of our pre-set size and circularity limits to ensure we did not unintentionally filter out a significant number of spores or cells. The most common particle is the single pixel artifact, which is caused by noise. Other small particles like the ones shown in Fig. 6 have sizes of $\le$ 50 pixels and have low circularity. These particles usually consist of segmented debris or partially obscured cells. Large filtered particles, such as spore particles greater than 750 pixels (2.7 $\upmu$m^2^) in size, consist of 2–3 cells or spores in close proximity, fused into a single particle by a pixel bridge (Fig. S4). However, these were relatively rare, accounting for only 0.1% and 0.5% of the cells in the 0 min and 120 min images, respectively. Therefore, they do not affect the results or capability of the workflow. These fused cells can also be resolved back into single images using the erode and dilate tools in ImageJ, although this would slightly affect their size.

Based on the information obtained, we were able to calculate the spore germination rate. After analysing the samples post-incubation, we identified 9455 segmented cells in the sample, of which only 1247 met the predefined criteria for spore size and circularity parameters before incubation. Using these results, we estimate the germination rate at 87%. By utilizing this approach, which enables scanning of large areas to inspect thousands of cells, it is possible to conduct various types of quantitative studies, including those focused on comparing growth rates or viability (live-dead) assays. As a proof of concept, we tested our UNet algorithm on images that contained spores exposed to high levels of 1064 nm light. See the method in the supplementary material. Thus, we expected that the majority of spores would be non-viable and that there would be fewer cells overlapping. In Fig. S6A and B, we can see that after 180 minutes of incubation, only a low number of spores (16) germinated out of a population of 633 (2.5% viability). These germinated spores can be clearly identified based on their size (Fig. S6C). The size and circularity (Fig. S6D) of the remaining population are similar between the start and end of the experiment (180 min).

These findings demonstrate that our algorithm provides a powerful tool for quantifying spore disinfection at the single-spore level by enabling high-throughput analysis of thousands of individual cells. By applying strict morphological criteria such as size and circularity, we can accurately distinguish viable, germinated spores from non-viable ones. The algorithm’s ability to detect such low levels of viability highlights its sensitivity and potential for evaluating disinfection efficacy in various experimental conditions.

### GUI application

We have developed a desktop application using Python’s Flask framework, designed to simplify the process of uploading and analyzing images with our automated algorithm. The application’s workflow is shown in Fig. S5. Users can select an image from a gallery or upload one via drag-and-drop. Once an image is uploaded, the application predicts the presence of spores. When installed on a local computer with an i9 10th Gen processor (Intel) and 32GB of RAM, the application processes both small and large images efficiently.

#### Github resources

Detailed instructions for installing the developed code and implementing it are provided in the supporting information. Additionally, users can find instructions on how to retrain using Google Colab in the GitHub resources^45^. The ’Training$\_$model.ipynb’ file can be used to retrain the model; simply follow the instructions provided in the file. The ’prediction$\_$file.ipynb’ file is used to test the model on your custom dataset.

## Conclusions

Light microscopy is a highly flexible tool for analyzing cell shape and the growth of cells. However, traditional methods often encounter challenges when scaling effectively for large numbers of cells. In this study, we developed an attention-based UNet algorithm specifically designed to rapidly analyze large microscope images, capable of accommodating more than 10,000 bacterial cells per image at each time point. Furthermore, we show a workflow that includes automated image acquisition over an extensive grid, followed by stitching these images into a larger, combined image. This approach presents significant advantages over conventional bulk methods in analyzing extensive cell populations. We believe that this workflow, coupled with the attention-based UNet algorithm, has substantial potential for a wide range of research applications, such as decontamination studies and live-dead assays.

## Supplementary Information

Supplementary Information.

## Data Availability

All the code in this project was developed using Python and different public libraries, as defined in the supporting information. The code can be downloaded from GitHub.

## References

[1] Stephens, D. J. & Allan, V. J. Light microscopy techniques for live cell imaging. *Science* **300**, 82–86 (2003). 12677057 10.1126/science.1082160

[2] Raguse, M. et al. Improvement of biological indicators by uniformly distributing Bacillus subtilis spores in monolayers to evaluate enhanced spore decontamination technologies. *Appl. Environ. Microbiol.* **82**, 2031–2038 (2016). 26801572 10.1128/AEM.03934-15 PMC4807530

[3] Görgüç, A. et al. Single and combined decontamination effects of power-ultrasound, peroxyacetic acid and sodium chloride sanitizing treatments on Escherichia coli, Bacillus cereus and Penicillium expansum inoculated dried figs. *LWT* **140**, 110844 (2021).

[4] Malyshev, D. et al. Hypervirulent R20291 Clostridioides difficile spores show disinfection resilience to sodium hypochlorite despite structural changes. *BMC Microbiol.* **23**, 59 (2023). 36879193 10.1186/s12866-023-02787-z PMC9986864

[5] Young, J. W. et al. Measuring single-cell gene expression dynamics in bacteria using fluorescence time-lapse microscopy. *Nat. Protoc.* **7**, 80–88 (2012). 10.1038/nprot.2011.432 PMC4161363 22179594

[6] Lisle, J. T., Hamilton, M. A., Willse, A. R. & McFeters, G. A. Comparison of fluorescence microscopy and solid-phase cytometry methods for counting bacteria in water. *Appl. Environ. Microbiol.* **70**, 5343–5348 (2004). 15345419 10.1128/AEM.70.9.5343-5348.2004 PMC520900

[7] Jeckel, H. & Drescher, K. Advances and opportunities in image analysis of bacterial cells and communities. *FEMS Microbiol. Rev.* **45**, 1–14 (2021). 10.1093/femsre/fuaa062 PMC8371272 33242074

[8] Hartmann, R., van Teeseling, M. C. F., Thanbichler, M. & Drescher, K. BacStalk: A comprehensive and interactive image analysis software tool for bacterial cell biology. *Mol. Microbiol.* **114**, 140–150 (2020). 32190923 10.1111/mmi.14501

[9] Jiang, J. et al. Segmentation, tracking, and sub-cellular feature extraction in 3D time-lapse images. *Sci. Rep.* **13**, 3483 (2023). 36859457 10.1038/s41598-023-29149-z PMC9977871

[10] Antonelli, L. et al. ALFI: Cell cycle phenotype annotations of label-free time-lapse imaging data from cultured human cells. *Sci. Data* **10**, 677 (2023). 37794110 10.1038/s41597-023-02540-1 PMC10551030

[11] Pandey, R. et al. Live cell imaging of germination and outgrowth of individual Bacillus subtilis spores; the effect of heat stress quantitatively analyzed with SporeTracker. *PLoS ONE* **8**, e58972 (2013). 23536843 10.1371/journal.pone.0058972 PMC3607599

[12] Berg, S. et al. Ilastik: Interactive machine learning for (Bio)image analysis. *Nat. Methods* **16**, 1226–1232 (2019). 31570887 10.1038/s41592-019-0582-9

[13] Bannon, D. et al. DeepCell Kiosk: Scaling deep learning-enabled cellular image analysis with Kubernetes. *Nat. Methods* **18**, 43–45 (2021). 33398191 10.1038/s41592-020-01023-0 PMC8759612

[14] Stringer, C., Wang, T., Michaelos, M. & Pachitariu, M. Cellpose: A generalist algorithm for cellular segmentation. *Nat. Methods* **18**, 100–106 (2021). 33318659 10.1038/s41592-020-01018-x

[15] Cutler, K. J. et al. Omnipose: A high-precision morphology-independent solution for bacterial cell segmentation. *Nat. Methods* **19**, 1438–1448 (2022). 36253643 10.1038/s41592-022-01639-4 PMC9636021

[16] Qamar, S., Öberg, R., Malyshev, D. & Andersson, M. A hybrid CNN-random forest algorithm for bacterial spore segmentation and classification in TEM images. *Sci. Rep.* **13**, 18758 (2023). 37907463 10.1038/s41598-023-44212-5 PMC10618482

[17] Ronneberger, O., Fischer, P. & Brox, T. U-net: Convolutional networks for biomedical image segmentation. In *Medical Image Computing and Computer-Assisted Intervention–MICCAI 2015: 18th International Conference, Munich, Germany, October 5-9, 2015, Proceedings, Part III 18* (Springer) pp 234–241 (2015).

[18] Wu, J. et al. A state-of-the-art survey of U-Net in microscopic image analysis: From simple usage to structure mortification. *Neural Comput. Appl.* **36**, 1–30 (2023).

[19] Panigrahi, S. et al. Misic, a general deep learning-based method for the high-throughput cell segmentation of complex bacterial communities. *Elife* **10**, e65151 (2021). 34498586 10.7554/eLife.65151 PMC8478410

[20] Qamar, S., Ahmad, P. & Shen, L. Dense encoder-decoder-based architecture for skin lesion segmentation. *Cogn. Comput.* **13**, 583–594 (2021).

[21] Valanarasu, J. M. J., Sindagi, V. A., Hacihaliloglu, I. & Patel, V. M. Kiu-net: Overcomplete convolutional architectures for biomedical image and volumetric segmentation. *IEEE Trans. Med. Imaging* **41**, 965–976 (2021). 10.1109/TMI.2021.3130469 34813472

[22] Niu, Z., Zhong, G. & Yu, H. A review on the attention mechanism of deep learning. *Neurocomputing* **452**, 48–62 (2021).

[23] Oktay, O., Schlemper, J., Folgoc, L. L, Lee, M., Heinrich, M., Misawa, K., Mori, K., McDonagh, S., Hammerla, NY., Kainz, B. *et al.* Attention U-net: Learning where to look for the pancreas (2018). arXiv preprint arXiv:1804.03999.

[24] Wu, H., Zhao, Z. & Wang, Z. META-Unet: Multi-scale efficient transformer attention Unet for fast and high-accuracy polyp segmentation. *IEEE Trans. Autom. Sci. Eng.* **21**, 4117–4128 (2023).

[25] Lindsay, G. W. Attention in psychology, neuroscience, and machine learning. *Front. Comput. Neurosci.* **14**, 29 (2020). 32372937 10.3389/fncom.2020.00029 PMC7177153

[26] Oktay, O., Schlemper, J., Folgoc, L. L, Lee, M., Heinrich, M., Misawa, K., Mori, K., McDonagh, S., Hammerla, NY., Kainz, B. *et al.* Attention u-net: Learning where to look for the pancreas (2018). arXiv preprint arXiv:1804.03999.

[27] Schlemper, J. et al. Attention gated networks: Learning to leverage salient regions in medical images. *Med. Image Anal.* **53**, 197–207 (2019). 30802813 10.1016/j.media.2019.01.012 PMC7610718

[28] Chen, H. & Murphy, R. F. Evaluation of cell segmentation methods without reference segmentations. *Mol. Biol. Cell* **34**, ar50 (2023). 36515991 10.1091/mbc.E22-08-0364 PMC10208095

[29] Peng, L., Chen, D., Setlow, P. & Li, Y. Q. Elastic and inelastic light scattering from single bacterial spores in an optical trap allows the monitoring of spore germination dynamics. *Anal. Chem.* **81**, 4035–4042 (2009). 19374431 10.1021/ac900250x PMC2717560

[30] Malyshev, D., Robinson, N. F., Öberg, R., Dahlberg, T. & Andersson, M. Reactive oxygen species generated by infrared laser light in optical tweezers inhibits the germination of bacterial spores. *J. Biophotonics* **15**, 1–7 (2022). 10.1002/jbio.202200081 35538633

[31] Andersson, M., Fällman, E., Uhlin, B. E. & Axner, O. Dynamic force spectroscopy of E. coli P pili. *Biophys. J .* **91**, 2717–2725 (2006). 16844748 10.1529/biophysj.106.087429 PMC1562381

[32] Stangner, T. et al. Cooke-Triplet tweezers: more compact, robust, and efficient optical tweezers. *Opt. Lett.* **43**, 1990 (2018). 29714728 10.1364/OL.43.001990

[33] Shorten, C. & Khoshgoftaar, T. M. A survey on image data augmentation for deep learning. *J. Big Data* **6**, 1–48 (2019). 10.1186/s40537-021-00492-0 PMC8287113 34306963

[34] Dwyer, B., Nelson, J., Solawetz. J. et al. Roboflow (version 1.0) [software] Available from https://roboflow.com computer Vision (2022).

[35] Vujović, Z. Classification model evaluation metrics. *Int. J. Adv. Comput. Sci. Appl.* **12**, 599–606 (2021).

[36] Malyshev, D. et al. Mode of action of disinfection chemicals on the bacterial spore structure and their Raman spectra. *Anal. Chem.* **93**, 3146–3153 (2021). 33523636 10.1021/acs.analchem.0c04519 PMC7893628

[37] Schindelin, J. et al. Fiji: An open-source platform for biological-image analysis. *Nat. Methods* **9**, 676–682 (2012). 22743772 10.1038/nmeth.2019 PMC3855844

[38] Preibisch, S., Saalfeld, S. & Tomancak, P. Globally optimal stitching of tiled 3D microscopic image acquisitions. *Bioinformatics* **25**, 1463–1465 (2009). 19346324 10.1093/bioinformatics/btp184 PMC2682522

[39] Kingma, D. P., & Ba, J. Adam: A method for stochastic optimization arXiv:1412.6980 (2014).

[40] Fawley, W. N. et al. Efficacy of hospital cleaning agents and germicides against epidemic clostridium difficile strains. *Infect. Control Hosp. Epidemiol.* **28**, 920–925 (2007). 17620238 10.1086/519201

[41] Ikram, S. et al. Bacillus cereus biofilm formation on central venous catheters of hospitalised cardiac patients. *Biofouling* **35**, 204–216 (2019). 30950292 10.1080/08927014.2019.1586889

[42] Shaheen, R., Svensson, B., Andersson, M. A., Christiansson, A. & Salkinoja-Salonen, M. Persistence strategies of Bacillus cereus spores isolated from dairy silo tanks. *Food Microbiol.* **27**, 347–355. 10.1016/j.fm.2009.11.004 (2010). 20227599 10.1016/j.fm.2009.11.004

[43] Gemein, S. et al. Efficacy of five ‘sporicidal’ surface disinfectants against Clostridioides difficile spores in suspension tests and 4-field tests. *J. Hosp. Infect.* **122**, 140–147 (2022). 35077809 10.1016/j.jhin.2022.01.010

[44] 2023 Guidance on the BPR: Volume II Efficacy, Assessment + Evaluation (Parts B+C) Tech. rep. European Chemicals Agency https://echa.europa.eu/guidance-documents/guidance-on-biocides-legislation.

[45] Qamar, S. Accessed 2025-05-01 Sporeunet [model code] https://github.com/sqbqamar/SporeUNet computer Vision.

