# Supplementary material for: Attention-driven UNet enhancement for accurate segmentation of bacterial spore outgrowth in microscopy images
Source: Sci Rep. 2025 Jun 20;15:20177. doi: 10.1038/s41598-025-05900-6 (PMC12181384; doi:10.1038/s41598-025-05900-6)
Supplement: Supplementary file 1 — Supplementary Information. [file 41598_2025_5900_MOESM1_ESM.pdf]

Attention-Driven UNet Enhancement for Accurate Segmentation of Bacterial Spore Outgrowth in Microscopy Images

Saqib Qamar[1,2,4], Dmitry Malyshev[1], Rasmus Öberg[1], Daniel P.G. Nilsson[1], Magnus Andersson[1,2,4]

[1]Department of Physics, Umeå University, Umeå, Sweden
[2]Integrated Science Lab, Department of Physics, Umeå University, Sweden
[3]Umeå Centre for Microbial Research (UCMR), Umeå, Sweden
[4]Department of Computing and IT, Sohar University, Sohar, Oman

Contents

Supporting figures........2
Quick guide to use the model on a Local computer........5
How to use the model on GitHub with Binder........6
How to use web-based application (Flask API)........8
Image Acquisition in LabView........9
Image stitching in ImageJ........10

Supporting figures

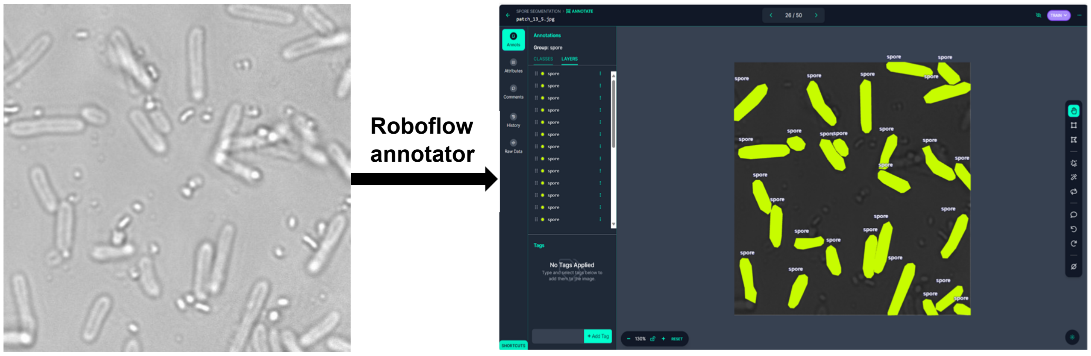

Figure S1 The Roboflow annotation software was utilized for image annotation, where polygon points are drawn over the area of the object to delineate its shape. However, some segmentations were not accurately corrected, and manual adjustment using polygon points was required for improved accuracy.

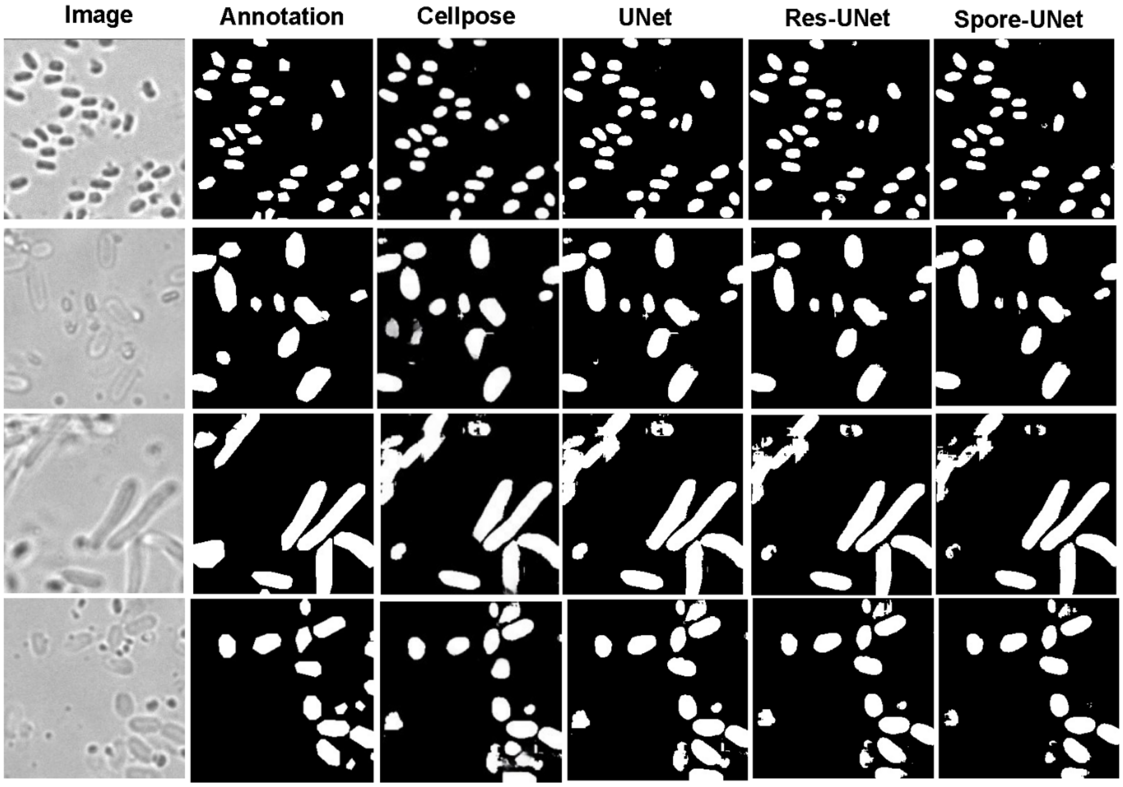

Figure S2 Example images showing additional examples of predictions, with the original images, annotated images, and the prediction made by the spore-UNet, Res-Unet, UNet, and Cellpose. In the panels both spores, vegetative growing cells, and debris are seen. Scale bar is 4 μm.

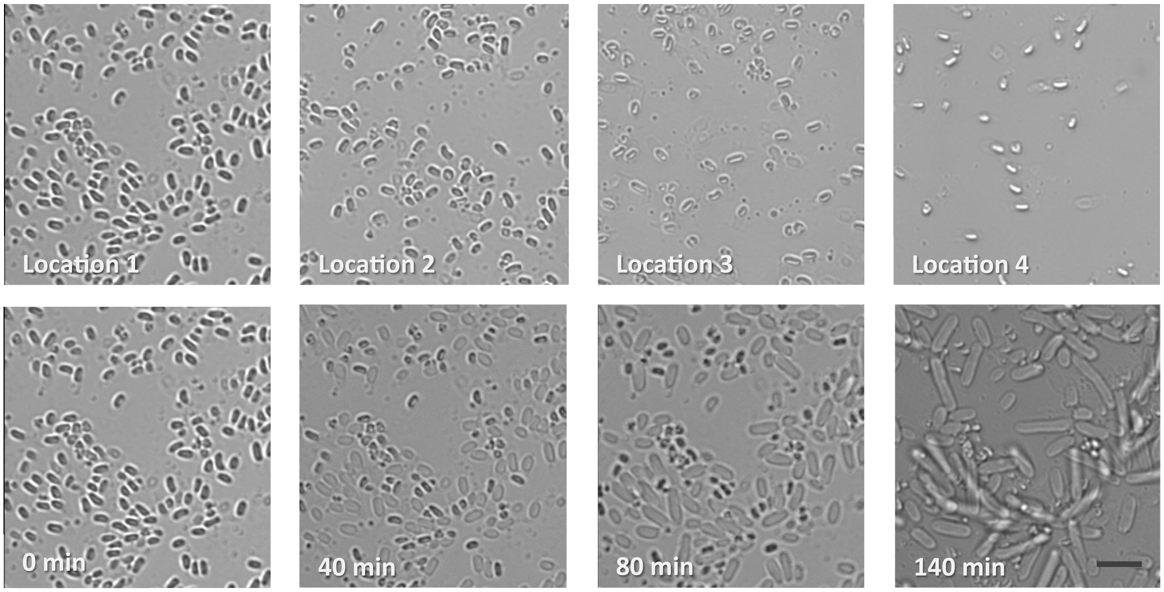

Figure S3 Factors germinating and growing bacterial spores that can affect automatic segmentation. All images are from the same sample. The first row highlights the differences in spore appearance due to different focus, with spores looking dark in location 1, but transitioning to low contrast grey in location 3, to bright in the location 4. It can be a big challenge for an algorithm to recognize both as the same object type. The second row shows the germination and growth of spores, which changes their size, shape and optical density as well as overlapping cells. Scale bar is 4 µm.

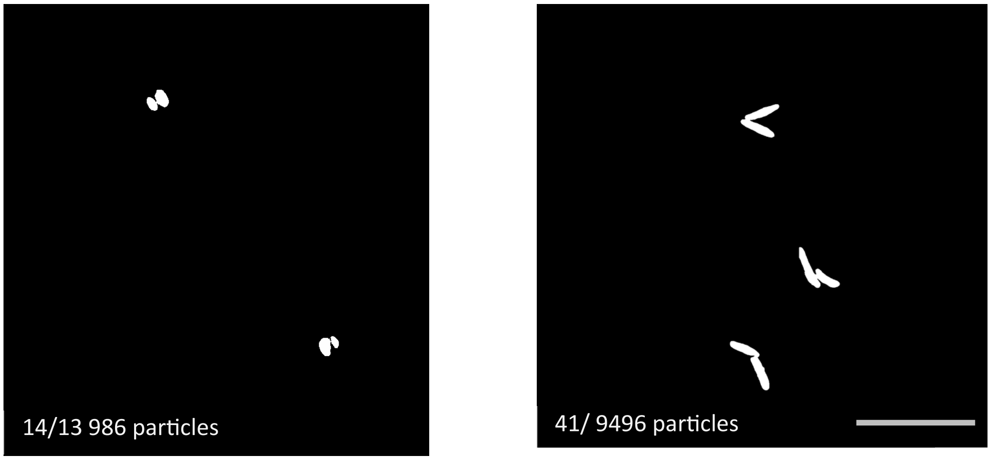

Figure S4 An uncommon issue in segmentation involves combining two or more distinct objects into a single entity. This issue was addressed by filtering out these merged objects based on their size and shape, particularly focusing on circularity. Such occurrences were relatively infrequent. For example, these two examples show that only 14 of 13986 particles and 41 of 9496 were identified as merged. The scale bar is 10 µm.

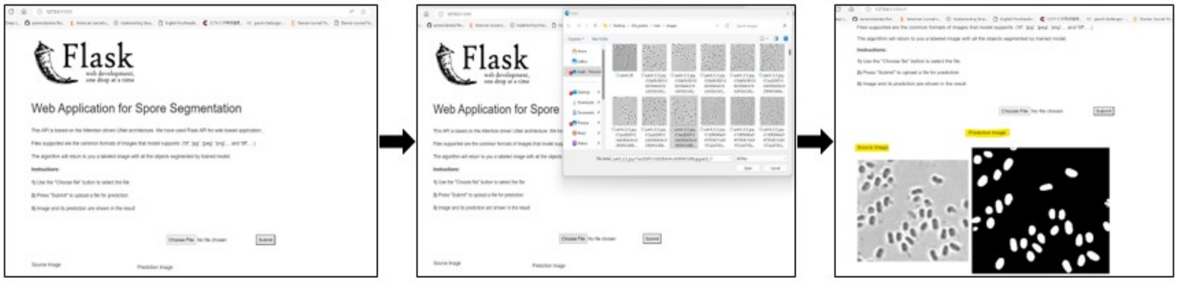

Figure S5. The image shows the different steps (left to right) of using the Flask based web application to get a prediction from the original image. You should try to send JPG format because of less memory consumption. If you want to convert a TIF file to JPG format, then a program file can be found in our GitHub repository.

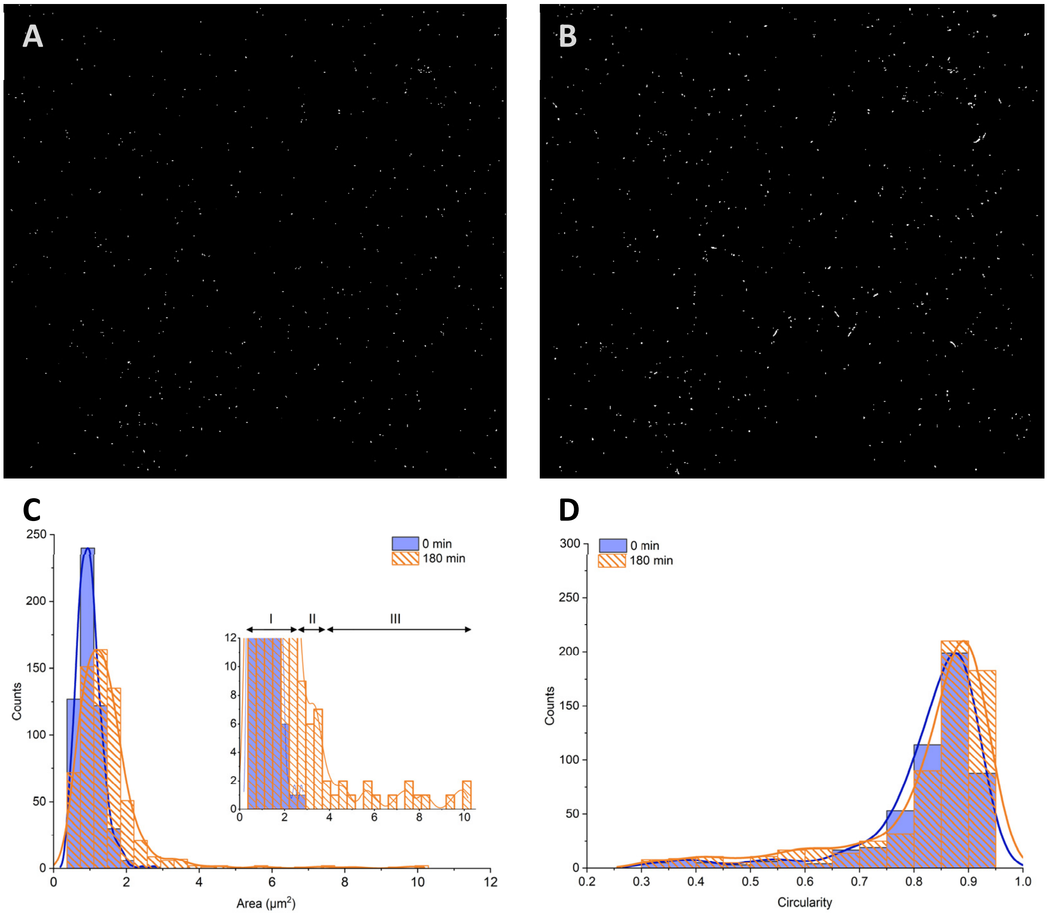

Figure S6. Tracking the growth and germination of inactivated *B. thuringiensis*. Similar to the images in Figure 7, the data is segmented at the start (A) and after 3 hours (B). Since only a small percentage of spores germinate, particle sizes remain largely unchanged (C and inset I), with some particles increasing slightly in size due to the hydration of spores (C, II), while the outgrown spores represent a small number of larger particles (C, III). Circularity (D) remained largely unaffected.

Spore decontamination method

B. thuringiensis spores were dried on a glass slide and illuminated with a pulsed 1064 nm laser light (80 ps pulses). In total, a 1.1 cm^2 area was being illuminated with a total 230 J of energy. The spores subsequently had 5 µL of tryptic soy broth added and were incubated for 3 hours at 30 °C as described in the methods.

Quick guide to use the model on a Local computer

Prerequisites:
- Conda (https://conda.io/)
- Code repository: https://github.com/sqbqamar/SporeUNet

Installation:
1. Download and extract the sporeUNet repo to a folder of choice.
2. Open a terminal at or navigate to the repo folder ("…/spore-main/").
3. Create and activate the conda environment with:

```
conda env create -f spore_env.yml
conda activate spore
```

Usage:
- Flask (Web app):
  1. Run:
     ```
     python app.py
     ```
  2. Open a web browzer and paste http://0.0.0.0:5000/ in the address bar.
  3. Follow the instructions on screen.

- Command line:
  1. As a demo, run:
     ```
     python cmd_prediction.py
     ```

This will run the prediction, segmentation with an input image from the repo and default parameters.

  2. Run the "cmd_prediction.py" python script with specific arguments.
  Example:

```
python cmd_prediction.py path/to/input/image.jpg path/to/output/directory
```

See output in the output folder.

How to use the model on GitHub with Binder

To use Jupyter Notebook **(prediction_file.ipynb)** on GitHub with a Binder link, follow these step-by-step instructions:

Access GitHub Repository:

Start by opening GitHub repository using a github link https://github.com/sqbqamar/SporeUNet

Click Binder Link:

a. Locate the Binder link which is available in README. It typically appears as a badge.
b. Click on the Binder link. This link will take you to the Binder service.

Binder Environment Setup:

After clicking the Binder link, Binder will automatically set up a computing environment for Jupyter Notebook. This environment includes the required libraries and packages. This process may take a few moments.

Launch Jupyter Notebook:

Once the Binder environment is ready, it will open Jupyter Notebook (prediction_file.ipynb) in a web browser.

Check Model and Image Paths:

In Jupyter Notebook, the first thing you should do is ensure that the paths to trained model file and image are correctly set. You can typically find this information in the first few code cells of the notebook. Verify that the paths are accurate.

Explore Cell-Wise Code:

You can now explore Jupyter Notebook cell by cell. This is where you will find the code for loading the trained model, passing input data (an image), making predictions, and displaying the results. Examine the code and comments within each cell to understand how the model is used for predictions.

View the Results:

After you've gone through the cells and made any necessary adjustments to paths or parameters, you can run the cells that perform predictions.

a. Execute the code cells to load the model, pass an input image, and generate predictions.
b. View the results, which visualizes in terms of bounding-box and mask on image.

Note: It Is Slow Loading

Environment Creation and Launching Kernel

If the loading time for Jupyter Notebook on Binder is slow, there are a few factors to consider:

- **Binder Server Load:** Binder relies on shared computing resources, and the server load can affect the speed. It is slower during peak times when many users are requesting Binder environments.

- **Internet Connection:** The user's internet connection also impacts loading times.

Speed Performance:

Unlike GPU, which is designed for parallel processing and is highly efficient for deep learning, CPU is slower in handling computations. So, notebook relies heavily on CPU-based calculations, which can impact processing speed.

How to use web-based application (Flask API)

Here is a step-by-step guide for using a web-based application to make predictions on a given input image using the trained model:

1. Clone or download the SporeUNet repository from GitHub (https://github.com/sqbqamar/SporeUNet)

2. Ensure you have the required packages installed (Keras, Tensorflow, flask, etc). You can install missing packages using pip.
3. Open the **app.py** file in an editor. Set the correct path for the model architecture (model_architecture.h5) and weights (spore_test_dec5.hdf5) files.
4. Open a terminal/command prompt and navigate to the app.py file location.
5. Type the following command to run the flask application:

python app.py

6. The application will start running on port 5000 and you will see the output:

Running on http://0.0.0.0:5000/

7. Open a web browser and enter the URL http://0.0.0.0:5000/ to open the web application.
8. Upload an image you want to make predictions on.
9. Click on the "Predict" button. The output prediction will be displayed below the input image.
10. To exit the application, go to the terminal window and press Ctrl+C.

Image Acquisition in LabView

A pseudocode for the snake-by-rows image capturing algorithm. The ratio between image and step size dictates the image overlap and this should be set to include sufficient features for image stitching. The LabView VI (virtual instrument) implements this algorithm together with divers for Hamamatsu Video Capture, Physik Instrumente and proprietary servo controllers, which should be replaced with drivers for your specific hardware.

```
set currentFrameX & currentFrameY & directionX = 1

if ( currentFrameX < gridSizeX )            // Move one frame in alternating X directions
        currentFrameX ++
        moveX ( directionX * stepSizeX )
        saveImage
else
        directionX *= -1
        currentFrameX = 1

        if ( currentFrameY < gridSizeY )    // Move down one frame in Y direction until..
                currentFrameY ++
                moveY ( stepSizeY )
                saveImage
        else                                // ..acquisition complete
                return
        end if
end if
```

Image stitching in ImageJ

This method uses ImageJ (Fiji), which can downloaded from:

https://fiji.sc/

The stitching plugin is also required

https://imagej.net/plugins/image-stitching

1. Open the stitching plugin under Plugins -> Stitching.

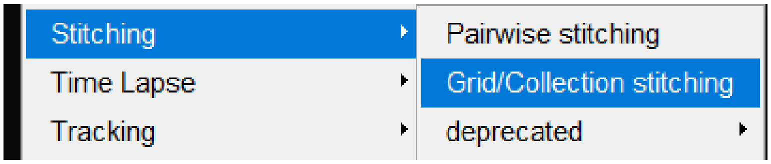

Open the grid/collection stitching if using more than 2 images.

2.

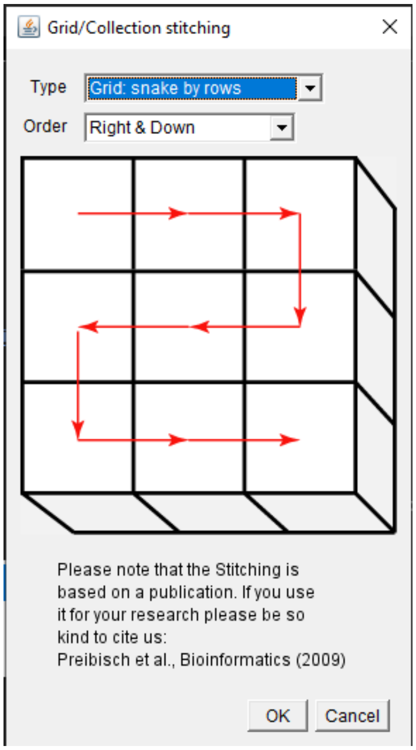

Use grid snake by rows (that's what the image arrangement in the laser power dataset). Other ways of tiling are available.

Note the citation below if you use the script in data that is published.

3.

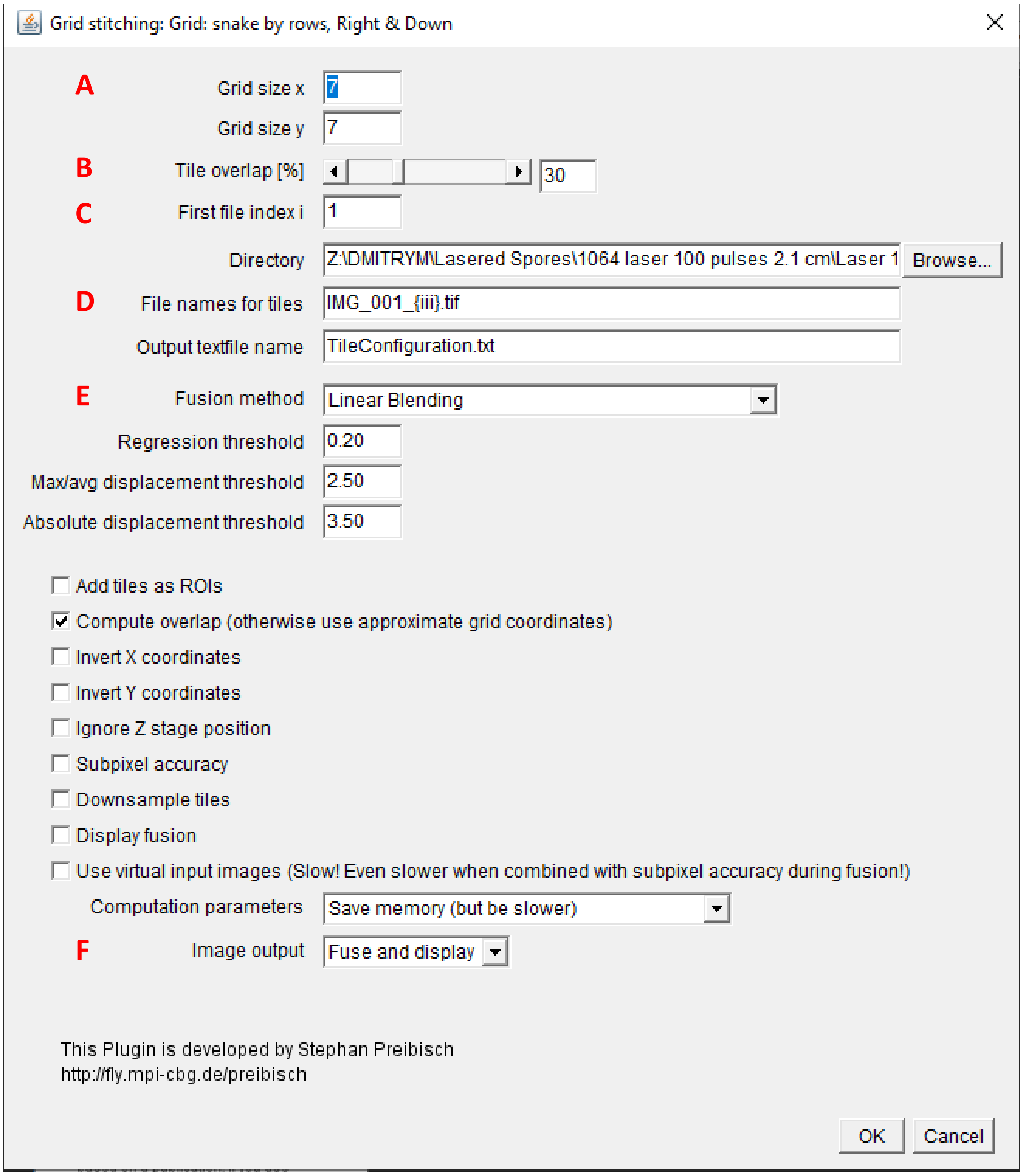

The screenshot above shows the grid menu with filled-in settings that results in an acceptable fused image in the test data.

A. Grid size. The data in this work data is 7x7

B. Tile overlap is an approximate number of how much overlap there is between different images. Note that the non-edge tile are overlapped on multiple sides, so this number may need to be set higher than the initial estimate

C. First file index. If first file increment starts with a 1, then it's 1. Usually it is the easiest way to do things, however, if you have multiple time points with incremental file names, then adjust the numbers as needed.

D. File names, the incrementing part of the file is replaced with {i}:
The first file name in the folder was: IMG_001_001.tif
So what you write into the window is:IMG_001_{iii}.tif

E. Fusion method. Recommend Linear blending produces a seamless combined image. Max/min intensity allows you to see the tiles to check if they are stitched well in terms of borders. Recommend doing a least once to check that the parameters are good.

regression threshold: if you set this number high, it may fail to stitch images properly and assume they are non-overlapping.
max/avg displacement threshold: for post-stitching optimisation. Larger numbers give some more leeway to adjust individual tiles.
absolute displacement threshold: Normally doesn't need to be changed, as it can break the image sequence by thinking images are not linked

F. Either computational method works, and both will be done in <1 min on a work computer.

The output image after these steps is shown on the next page:

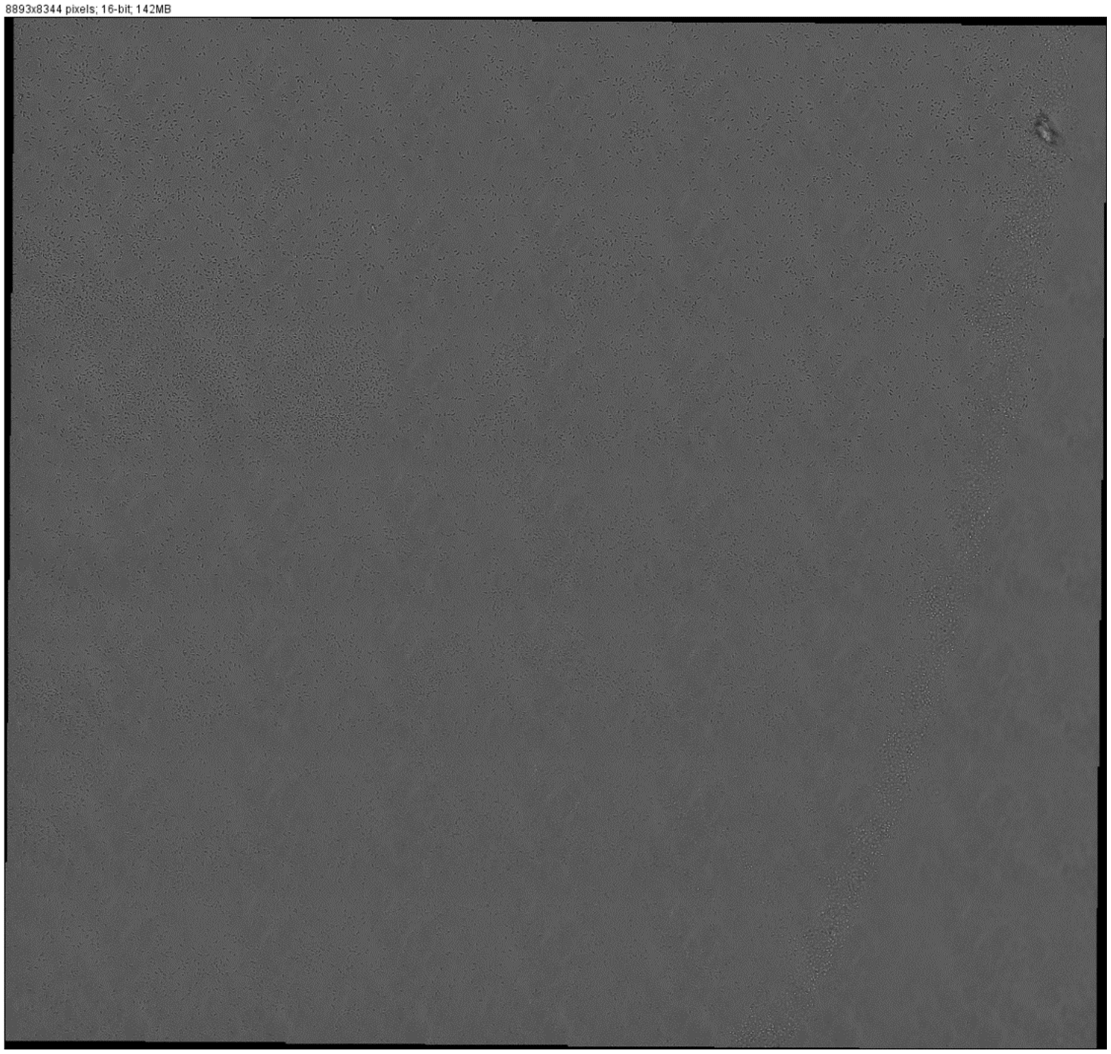

If the stitching does not produce a good image (wrong shape, poor overlap), check the tile overlap and regression threshold parameters.

A more detailed guide is available here:

https://imagej.net/plugins/image-stitching
